# Myasthenia Gravis: A Review of Available Treatment Approaches

**DOI:** 10.4061/2011/847393

**Published:** 2011-10-05

**Authors:** Nils Erik Gilhus, Jone F. Owe, Jana Midelfart Hoff, Fredrik Romi, Geir Olve Skeie, Johan A. Aarli

**Affiliations:** ^1^Department of Clinical Medicine, University of Bergen, 5020 Bergen, Norway; ^2^Department of Neurology, Haukeland University Hospital, 5021 Bergen, Norway

## Abstract

Patients with autoimmune myasthenia gravis (MG) should be further classified before initiating therapy, as treatment response varies for ocular versus generalised, early onset versus late onset, and acetylcholine receptor antibody positive versus MuSK antibody positive disease. Most patients need immunosuppression in addition to symptomatic therapy. Prednisolone and azathioprine represent first choice drugs, whereas several second choice options are recommended and should be considered. Thymectomy should be undertaken in MG with thymoma and in generalised, early-onset MG. For MG crises and other acute exacerbations, intravenous immunoglobulin (IvIg) and plasma exchange are equally effective and safe treatments. Children and females in child bearing age need special attention regarding potential side effects of immunosuppressive therapy. MG pathogenesis is known in detail, but the immune therapy is still surprisingly unspecific, without a pin-pointed attack on the defined disease-inducing antigen-antibody reaction being available.

## 1. Introduction

Myasthenia gravis (MG) has a prevalence of 150 per million, with nearly one million MG patients worldwide. The yearly incidence is 10–15 per million per year [1]. Before any treatment was available the prognosis was severe, with an expected 50% 10-years' mortality. With modern treatment facilities such as immunotherapy, thymectomy, and intensive care facilities available, population-based studies show that MG and non-MG individuals have the same life expectancy [2], but still often with reduced physical abilities, reduced quality of life, and risk of complications. 

There are three key aspects of MG which define the therapeutic opportunities.

- MG is a well-defined autoimmune disease and thus responds to immunoactive treatment.
- MG is caused by impaired acetylcholine receptor (AChR) stimulation in the postsynaptic skeletal muscle membrane and thus responds to an increase in AChR activity.
- MG has muscle weakness as the only symptom, and consequently should respond to measures that increase muscle function and counteract muscle weakness.

MG treatment is firmly established as the domain of neurologists. Neurologists should be in charge even if the target organ is skeletal muscle, disease mechanisms are systemic, thymus is a target organ for diagnostic, therapeutic and scientific approach, hypoventilation is a life-threatening symptom, and diplopia often the most troublesome symptom. Ten percent of MG patients have another autoimmune disorder in addition, further supporting the need for complementary medical competence. Close cooperation with other fields of medicine provides knowledge regarding new immunoactive drugs, thus expanding the therapeutic opportunities for MG. 

For complicated and rare disorders such as MG, the establishment of medical centres supervising the treatment of the majority of MG patients and of all complicated patients is important to improve treatment quality. Increased treatment experience will optimize present therapy and facilitate the introduction of new and better treatment procedures. Centres with special competence and qualifications in MG treating the majority of patients will further enhance research, including well-controlled and prospective treatment studies. 

Ideally treatment recommendations should be based on scientific evidence of high quality, preferentially more than one blinded and controlled prospective study with a sufficient number of well-defined MG patients. There are disappointingly few such studies for MG. Recommendations therefore, rely on studies of lower quality and even sometimes only on case reports, clinical experience, and knowledge from non-MG treatment. It is important for patients as well as doctors to know which treatment is supported by high-quality evidence and which is more tentative and based on clinical experience and circumstantial evidence.

## 2. MG Classification

The various subgroups of autoimmune MG respond differently to treatment. Thus, before deciding any treatment, all individual MG patients should be defined according to subgroups. Classification aspects reflect the investigations of each patient that are necessary to undertake [3]:

1. early-onset MG: age at onset <50 years. Thymic hyperplasia;
2. late-onset MG: age at onset >50 years. Thymic atrophy;
3. thymoma-associated MG;
4. MG with anti-MuSK antibodies;
5. ocular MG: symptoms only from periocular muscles;
6. MG with no detectable AChR and MuSK antibodies;

The MG group with no detectable antibodies is heterogeneous. Some of these patients have low-affinity AChR antibodies that are not detectable by the routine assays and sometimes also thymic hyperplasia [4]. Some may similarly have undetectable MuSK antibodies, and some most probably have autoantibodies against other antigen(s) in the postsynaptic membrane. There are not yet any commercial tests available for the low-affinity AChR antibodies [5]. MG patients with a thymoma have nearly always detectable AChR antibodies in serum. Necessary investigations include tests for AChR and MuSK autoantibodies and CT/MR of the anterior mediastinum. Titin and ryanodine receptor antibodies may be helpful for classification. For patients with no AChR and MuSK antibodies, it is necessary with thorough examinations to exclude other causes for their muscle weakness, including nonautoimmune myasthenic syndromes. Neurophysiological examinations with repetitive nerve stimulation and jitter measurements are important to establish the initial diagnosis, especially in patients without detectable antibodies.

MG should be classified according to severity [6]. This is important when deciding specific treatment in the individual patient. It is also important in the followup to evaluate effects of various interventions. An accurate MG severity evaluation is crucial for controlled therapeutic studies. MG represents a challenge for such evaluation due to variation among muscle groups and variation during the day.

MG in early childhood poses special treatment challenges linked to growth and development in general and of the immune system [7]. The same is true for treatment of MG women in childbearing age, mainly due to potential effects of the disease and the therapies on the developing child in utero [8]. Epidemiology differs between ethnic populations and also regarding the frequency of the various MG subgroups [9]. However, MG patients are classified in the same way universally. Nonautoimmune myasthenic syndromes (genetic, toxic) and non-MG autoimmune syndromes (LEMS, neuromyotonia) are not included in this review.

## 3. Treatment of Acute Exacerbations

Acute exacerbations of MG need effective and urgent life-saving treatment. Life-threatening hypoventilation is the utmost threat. Plasma exchange and intravenous immunoglobulin (IvIg) are both effective for acute MG [10]. Their beneficial and symptom-relieving effect is regarded as well proven from several studies and from widespread clinical use [11]. In contrast to most other treatment options, the clinical response is rapid, occurring already after 2-3 days and often with a dramatic effect. This treatment should be given for severe exacerbations and is mandatory for MG crisis or threatening crisis. Plasma exchange or IvIg can also be used for less severe exacerbations, before surgery or together with initiation of immunosuppressive therapy with a slower effect [12]. Severe MG exacerbations with impaired respiratory function need hospitalization and often intensive care treatment.

Plasma exchange and IvIg have a similar clinical effect, and a similar responder rate. The only controlled and randomised study did not show any difference for these two treatment options [13]. Also nonrandomised evidence favours an equal effect, although the clinical impression may be a somewhat faster and more extensive effect for plasma exchange. IvIg has less side effects and less severe side effects. Optimal technique and high experience reduce the complication rate, especially for plasma exchange. Both treatment options are expensive, but IvIg represents a simpler procedure and may be superior from a total economic perspective [11]. Patients responding to plasma exchange and IvIg are not necessarily the same. Thus, if one treatment fails, the other may well be tried. It should be more convenient to add IvIg after plasma exchange than doing the procedure the other way around, this is to avoid washing away all therapeutic immunoglobulin just given to treat the patient. 

For severe MG and in an acute situation, high-dose parenteral corticosteroids can be given and also in addition to plasma exchange or IvIg [10]. An early exacerbation can be seen after initiation of corticosteroids, but with pharmacological doses, a therapeutic effect often appears very early.

## 4. Drug Treatment

### 4.1. General

Patients with the diagnosis of MG should always be considered for symptomatic as well as immunoactive drug treatment. Nearly all patients need some treatment, at least in periods where the disease shows clinical activity with permanent or intermittent muscle weakness. Symptomatic drugs have a short-lasting activity both regarding effect and side effects. Dosage can be rapidly changed and the treatment is flexible. Immunoactive drugs have an effect linked to pathogenesis, and the effect usually needs some time before it becomes manifest. Side effects are relevant and should be considered in a long-term perspective. Immunoactive drugs need special attention in children and MG women of childbearing age [6]. Thus, the considerations for patient and doctor are different for symptomatic and immunoactive drug treatment.

### 4.2. Symptomatic Drugs

Acetylcholine esterase inhibition at the neuromuscular junction has a symptomatic effect in myasthenia and especially in autoimmune MG [3, 10, 14–16]. Optimal dosage is adjusted according to effect and side effects. Side effects appear from the nonneuromuscular cholinergic synapses in the autonomic system, which are overstimulated. Alternative ways to increase the amount of acetylcholine at the neuromuscular end plate have been tried, but with less effect than inhibiting the degradation. Acetylcholine esterase inhibitors have a stable and predictable effect, apparently unchanged over years. No scientific comparisons have been undertaken between the various esterase inhibitors. The most commonly used is pyridostigmine and also the faster acting neostigmine. Ambenonium is used in some countries. Some MG patients with anti-MuSK antibodies are hypersensitive to an increase in acetylcholine concentration.

### 4.3. Immunoactive Drugs

*Prednisone/prednisolone *remains a first-choice drug in MG [3, 14–18]. It has a well-proven positive effect experienced through decades of clinical practice in a high number of patients. However, there are no formal trials and no scientific comparisons with other drugs. Side effects occur in most patients, and they are usually of clinical significance. Prednisone/prednisolone is regarded to be safe in pregnancy. To reduce the amount of side effects, dosing the drug every second morning is usually advocated. Most patients keep a sufficient clinical effect on the MG symptoms with this regimen and with markedly less side effects. Patients often continue to do well on a very low every second day dose, but experience an exacerbation if taking this low dose away. We recommend cautiousness regarding MG patients doing well and being stable on prednisone/prednisolone in a low dose; continued long-term treatment may be necessary. 

*Azathioprine *is the other well-established first-choice immunoactive drug used for MG [3, 14–18]. This drug is often used in combination with prednisone/prednisolone. Formal scientific evidence for its effect in MG is lacking, but a controlled trial showing the superiority of the combination prednisolone—azathioprine over prednisolone alone is much cited [19]. Azathioprine is regarded as safe and with few side effects, also during long-term treatment. It is listed among drugs that should not be used in pregnancy, although formal evidence of teratogenic effects in MG patients is lacking. During the first few months of treatment, the numbers of leucocytes and leucocyte subgroups have to be counted weekly. The clinical effect of azathioprine is slow to appear. Improvement should not be expected to appear until after 3–6 months, and full effect of the drug first occurs after 1-2 years. This is a reason why azathioprine is usually combined with other immunoactive treatments, such as prednisone/prednisolone, and especially in the initial phase. Marked improvement on azathioprine is reported in 70–90% of MG patients in open series. 

*Mycophenolate mofetil *is regarded as an alternative drug for mild MG [3, 14–18]. However, after promising results in open MG patient treatment, randomized and controlled trials failed to confirm a positive effect [20]. The drug has few and mild side effects and is easy to use both for patients and doctors. Despite its limitations, mycophenolate mofetil is still regarded as an alternative drug for mild MG, whereas more severe MG is usually not treated by this drug because of the negative controlled trials. 

*Methotrexate *should be used only when first-choice immunosuppressive drugs do not have sufficient effect [3, 14–18]. Methotrexate has a good and proven effect for other autoimmune disorders, but is not formally tested for MG. Still it should be tried in selected MG patients with a marked functional deficit, partly because it is usually well tolerated. 

*Cyclosporine A *is an inhibitor of T cells and has well-documented immunosuppressive effects after organ transplantation. A controlled prospective study with a limited number of included patients proved the effect of this drug for generalised MG [21]. Due to the danger of side effects, cyclosporine is regarded as a second-choice immunoactive drug for moderate to severe MG not responding to azathioprine and prednisolone [3, 14–18]. 

*Rituximab* is a chimaeric monoclonal antibody that targets B lymphocytes through its binding to the CD 20 molecule. MG is a prototype of an antibody-mediated autoimmune disease, and so rituximab and B-cell depletion are a very promising treatment alternative. In a recent review by Benveniste and Hilton-Jones [22], the effect of rituximab in 53 MG patients was recorded, including patients with both AChR and MuSK antibodies. The authors concluded that markedly positive effects were observed with distinct improvement of severe symptoms. Rituximab should be reserved for patients with severe MG, where treatment with prednisolone and at least two other standard immunosuppressive drugs has failed. For milder MG, the risk of progressive multifocal leucoencephalopathy and other potential long-term side effects probably outweigh its therapeutic potential. This drug seems to be particularly useful for anti-MuSK MG. 

*Tacrolimus (FK506) *is a calcineurin inhibitor just as cyclosporine. The drug inhibits the proliferation of activated T lymphocytes, but also acts on ryanodine receptor-mediated calcium release from sarcoplasmic reticulum in muscle cells. The drug has shown a beneficial effect in MG, and it represents an alternative second-choice drug for moderate to severe MG [3, 14–18], perhaps especially for MG patients with ryanodine receptor autoantibodies [23]. 

Other drugs, such as cyclophosphamide and several new and selective immunosuppressive drugs, have probably a positive effect on MG, as they have on other autoimmune disorders. However, this effect has not been documented so far or is less well documented than for the above-mentioned drugs. Potential side effects are significant.

## 5. Thymectomy

*Thymectomy* should be undertaken in all the 10–15% of MG patients with a thymoma. MG improvement sometimes occurs in such patients, but less consistently than in patients with a hyperplastic thymus. The main reason for thymectomy in thymoma patients is to remove a potentially infiltrating tumour [24]. In some patients with no or very mild MG symptoms, a severe exacerbation of MG with an increase in AChR autoantibodies has been reported after the removal of a thymoma [25]. 

*Thymectomy* should always be considered as an early therapeutic measure in early onset MG with generalised symptoms [3, 10]. Many patients benefit considerably. Thymic hyperplasia with an enlarged thymus and numerous germinal follicles is associated with improvement after thymectomy. Although no blinded and fully controlled studies have been undertaken, scientific evidence and clinical experience undoubtedly confirm thymectomy as a therapeutic option [26]. A transsternal approach and video-assisted thoracoscopy appear equally effective [27]. Postoperative improvement occurs gradually after 2–24 months. Age alone should not decide thymectomy or not. Some of the MG patients that experience their first symptoms after the age of 50 years have a hyperplastic thymus and also other features in common with the early onset MG group. Such patients are expected to respond to thymectomy.

**Nonthymectomy**

Late-onset MG patients should also be considered for thymectomy, but thymectomy is undertaken only in a minority of them [3, 10, 26]. Early debut age within this late-onset group (<60 years) and thymic hyperplasia on MR/CT imaging favour thymectomy. Higher age, symptoms for a longer time period, atrophic thymus, and presence of non-AChR antibodies against titin and/or ryanodine receptor all count against thymectomy.

For MG with purely ocular symptoms, thymectomy is not recommended. For MG patients with anti-MuSK antibodies, the majority of evidence points to no therapeutic effect of thymectomy. Anti-MuSK MG is probably not linked to thymic pathology.

**Thymectomy**

Some MG patients have no detectable muscle antibodies at repeated testing and even with generalised myasthenic symptoms. As a proportion of such seronegative MG patients have low-affinity AChR antibodies, thymectomy should be an option also for patients in this group. For patients with generalised MG, onset at relatively low age, and with a hyperplastic thymus on imaging, thymectomy should in our opinion be performed.

Thymectomy should always be undertaken in hospital units with experience in this type of surgery. Neurologists should evaluate the patients immediately before surgery and continuously in the postoperative phase. The patients should be in a stable condition at the time of surgery, and the threshold for treatment with plasma exchange or IvIg preoperatively should be low. Such treatment will secure a fast recovery and counteract postoperative complications, most importantly from the respiratory system.

## 6. Accessory Treatment

During acute MG exacerbations, intensive care therapy with respiratory support is life saving. Infections should be treated rigorously. The marked reduction in MG mortality is for a large part due to modern intensive care therapy [2], although also pharmacological treatment and thymectomy represent cornerstones in MG therapy. 

Physical training, weight control, and sensible life style modifications should be discussed with all MG patients [10]. Seasonal flu vaccination should be recommended. Symptomatic ophthalmological treatment may be helpful for ocular MG with troublesome diplopia.

## 7. Treatment Principles

MG should be treated early and with vigour, after classification of subtype and MG severity. Moderate or severe myasthenic weakness represents an immediate and permanent challenge. Treatment at an early stage with thymectomy and/or immunoactive drugs improve long-term outcome. With a lack of initial response, it is not sufficient to have tried only 2-3 alternative drug options. Drugs can be combined. Longitudinal measurement of AChR antibodies can be helpful in evaluating treatment effect and also in the differentiation between MG and non-MG symptoms experienced by the patient [5]. The clinical response and evaluation is most important, but there tends to be a correlation between MG severity/activity and AChR antibody concentration in the individual patient. There are no studies systematically and prospectively examining the usefulness of repeated antibody examinations in established MG. Non-MG drugs given to patients with MG should always be checked for potential adverse neuromuscular effects.

Most MG patients are in the need of long-term therapy. For patients in a stable remission when on immunoactive drugs, a conservative policy regarding full drug withdrawal is recommended. A low-dose prednisolone, azathioprine, or other immunoactive drugs can in such patients be sufficient to maintain the stable condition, but also necessary to avoid new exacerbations. Younger patients in particular, not least after thymectomy, can obtain a full clinical remission without any need for continued drug treatment. Late-onset MG patients and thymoma MG patients usually need life-long treatment. 

In 10% of MG patients, the onset is before age 18 years [6]. The disease is very rare in infancy. In Asian populations, up to 50% of cases present in adolescence [9]. Most children with MG have AChR antibodies. Those without antibodies should be thoroughly checked for non-MG myasthenic syndromes. The response to thymectomy is usually very favourable, and thymectomy should be done early. Immunosuppressive drugs are used for moderate and severe cases in the same way as for adults, but chronic administration of such drugs in children usually leads to significant side effects. Children more often obtain full remission after thymectomy, so that withdrawal of immunosuppressive drugs should be tried after a couple of years, especially if a marked reduction of AChR antibody titre has occurred. 

Pregnancy and giving birth for MG women is usually uncomplicated, although operative intervention during birth (Caesarean section, forceps use) occurs more frequently than in controls, related to prolonged labor [28]. Anticholinesterase drugs and prednisolone are considered to be safe during pregnancy. Azathioprine and other immunosuppressive drugs should be withdrawn before a planned pregnancy and should be avoided in the fetal organ-developing period. Arthrogryposis occurs with increased frequency in children of MG mothers, caused by movement inhibition in utero due to transplacental transfer of mother's AChR antibodies and thereby reduced function of the fetal AChR [28, 29]. 10–15% of the children of MG mothers experience a transient neonatal MG, usually mild and lasting only a few days. This risk is influenced by AChR antibody subclass and antigen specificity [7]. Lactation is recommended by most neurologists irrespective of mother's immunosuppressive MG drug treatment. Previous thymectomy does not influence pregnancy and giving birth negatively and could have a protective effect on neonatal MG [8]. Father's MG is not known to have any influence on the child, apart from the increased risk for MG and other autoimmune disease due to genetic factors. 

MuSK-antibody associated MG has a phenotype that differs from non-MuSK MG [14]. The patients tend to have more severe symptoms, and the therapeutic response is more variable. Cholinesterase inhibitors should be tried, but considered less beneficial for this subtype. Most patient series report no confirmed effect of thymectomy. Immunosuppressive drugs should be tried for the same indications as in non-MuSK MG. Prednisolone/prednisone and azathioprine have lower success rate in patients with MuSK antibody MG. As patients with this MG subtype often have severe and progressive symptoms and from bulbar and respiratory muscles, effective and intense therapy is necessary. Most patients use corticosteroids. From many case reports, rituximab and plasma exchange seem to be important alternatives often used, and the same is probably true for IvIg [10, 11, 14, 22].

## 8. Future Treatment

It is a paradox that MG treatment is still so unspecific. MG is the best characterised autoimmune disease with well-defined pathogenetic antibodies that impair function through the destruction and inhibition of muscle cell AChR. Still our therapeutic immunosuppression is aimed at general mechanisms of the immune system. The external causes for MG are totally unknown, apart from those 10–15% of patients with a paraneoplastic condition linked to thymic neoplasia. 

The ultimate aim of eradicating MG by removing the cause of the disease seems still far away. 

Antigen-specific treatment of MG should be promising, and such strategies work in animal models. Induction of specific immunological tolerance to AChR or MuSK, shifting the immune response from harmful to nonharmful is theoretically possible [3, 30, 31]. Strategies involving antigen-presenting cells are considered for treatment of autoimmune disorders, and manipulation of this process could be antigen-specific. T-cell receptor vaccination may be less promising, since T-cell receptor usage is not very restricted in human MG [32]. A sensible approach would be to remove the pathogenic autoantibodies specifically, or remove the plasma cells and/or B lymphocytes producing these antibodies [3]. So far such treatment has not been cost-effective. The effect has not been superior to today's standard treatment, costs have been higher, and procedures more complicated. Administering nonpathogenic AChR (or MuSK) antibodies to MG patients and thereby blocking the action of the patient's own pathogenic antibodies would be an alternative experimental approach. 

MG is improved by the inhibition of acetylcholine esterase. Other non-AChR molecules could theoretically be influenced therapeutically to improve the neuromuscular function. So far no such additional treatment has been established as effective in the clinical situation. 

New and more selective immunoactive drugs are marketed worldwide. These drugs are established as first- or second-line treatment for an increasing number of autoimmune and inflammatory disorders, due to their proven superior clinical effect. For MG, these drugs have not been evaluated in prospective and controlled trials. The present treatment has reasonably good effect in the large majority of MG patients, so that most patients have a high level of daily function and with few and modest side effects of the treatment. However, there is a need for better and more focused treatment. Such treatment should be established through formal multicenter trials in MG networks, not by random and individual off-label use of the drugs. As pathogenesis differs in MG subgroups, the immunoactive treatment needs to be individualised. Subgroups of MG will respond differently to various treatment alternatives. In the future, the detailed evaluation of each MG patient will hopefully have distinct therapeutic consequences, so that the treatment regime is tailored according to the specific autoimmune dysfunction.
